# Chemical Exposures: Genes and Sensitivity

**Published:** 2005-03

**Authors:** Angela Spivey

People who suffer from multiple chemical intolerances, a condition sometimes referred to as “multiple chemical sensitivity,” report wide-ranging symptoms such as headaches, short-term memory problems, confusion, fatigue, depression, irritability, and breathing difficulties. Is this phenomenon the result of a decreased physical tolerance to small amounts of chemicals such as pesticides and solvents, or is it an irrational fear of chemicals or a manifestation of psychological stress, as some have suggested? A study by University of Toronto epidemiologist Gail McKeown-Eyssen and her colleagues suggests that the condition may actually have a genetic basis.

The study, reported in the October 2004 *International Journal of Epidemiology*, investigated for the first time genetic differences between women reporting multiple chemical intolerances and those without such intolerances. Although both women and men report multiple chemical intolerances, several studies suggest that many more women than men may be affected.

The researchers recruited 203 cases and 162 controls from female respondents to a University of Toronto health survey. They identified multiple chemical intolerance sufferers using criteria derived from earlier studies, including one by James R. Nethercott and colleagues in the January/February 1993 *Archives of Environmental Health*, which defines cases as those with symptoms that are chronic, that are linked to a low level of exposure to chemical agents, and that resolve with removal of the exposure.

The Toronto researchers found that cases were significantly more likely than controls to exhibit specific polymorphisms in one or both of two genes, CYP2D6 and NAT2. CYP2D6 codes for enzymes that metabolize chemicals such as medications that target the central nervous system (including various antidepressants, stimulants, and codeine—all drugs with different chemical structures), drugs of abuse, neurotoxicants, procarcinogens (substances that become carcinogenic only when metabolized into more reactive compounds), and even the body’s own neurotransmitters. NAT2 also plays a role in metabolism of various drugs and toxic chemicals, including aromatic amines, a class of chemicals used in the production of epoxies and dyes.

Women whose polymorphism gave them higher CYP2D6 activity were more than three times more likely to be chemically intolerant than those with the inactive form of the gene. Likewise, women with the so-called rapid-acetylator form of NAT2 were four times more likely to report multiple chemical intolerances. Because metabolism of some chemicals can result in toxic by-products, people with rapid metabolisms could be more quickly accumulating toxic compounds in the body. “It depends on the compound, what the metabolites are, and how quickly they’re cleared from the body, as to whether having rapid metabolism results in more exposure or less exposure,” McKeown-Eyssen says.

The researchers found an even stronger association in women who showed the rapid-metabolizing form of both CYP2D6 and NAT2. These women were 18 times more likely than control subjects to suffer from multiple chemical intolerances. McKeown-Eyssen is particularly cautious about this finding because the analysis for such an interaction was not part of the original study design. “We have to be really careful about that observation,” she says. “But if it’s true and can be replicated, it means that some people are at very high risk.”

If replicated, these findings may provide evidence of a physical origin for this enigmatic disorder. It was only in 1994 that the American Medical Association acknowledged in a joint statement with other organizations that chemical intolerances should not be dismissed as psychogenic.

Claudia Miller, a professor of environmental medicine at the University of Texas Health Science Center at San Antonio, says that the three- and fourfold increases in risk associated with the polymorphisms are notable, and that the findings are important in suggesting a physical basis for these illnesses. “It’s hard to say that genetic polymorphisms are psychogenic,” she says. “For a long time we’ve known there’s a spectrum of [chemical intolerance] susceptibility in the population, and it should not be a surprise that it’s genetically based.”

McKeown-Eyssen says that after these results are replicated, further research should include studies of the function of the enzymes coded for by these genes. It might also be reasonable, she suggests, to look for other genes involved in chemical intolerance, since this study found an association when looking at only a few of the many genes involved in detoxification of chemicals.

